# Emerging approaches to study cell–cell interactions in tumor microenvironment

**DOI:** 10.18632/oncotarget.26585

**Published:** 2019-01-22

**Authors:** Nao Nishida-Aoki, Taranjit S. Gujral

**Affiliations:** ^1^ Division of Human Biology, Fred Hutchinson Cancer Research Center, Seattle, WA, USA

**Keywords:** cell–cell interaction, tissue heterogeneity, tumor microenvironment, single-cell analysis

## Abstract

Cell–cell interactions are of crucial importance for tissue formation, homeostasis, regeneration processes, and immune response. Recent studies underlined contribution of cell–cell interaction in tumor microenvironment (TME) for tumor progression and metastasis. Cancer cells modify the host cells to tumor-supportive traits, and the modified host cells contribute to tumor progression by interacting with cancer cells and further modifying other normal cells. However, the complex interaction networks of cancer cells and host cells remained largely unknown. Recent advances in high throughput microscopy and single cells-based molecular analyses have unlocked a new era for studying cell–cell interactions in the complex tissue microenvironment at the resolution of a single cell. Here, we review various model systems and emerging experimental approaches that are used to study cell–cell interactions focusing on the studies of TME. We discuss strengths and weaknesses of each model system and each experimental approach, and how upcoming approaches can solve current fundamental questions of cell–cell interactions in TME.

## CELL COMMUNICATION IN NORMAL PHYSIOLOGY AND IN CANCER

The human body is estimated to be composed of more than 200 different types of cells, with approximately 3.72 × 10^13^ cells in total [1]. Cells with specialized functions form functional units such as organs (brain, heart, liver, etc.), skin, bone, blood, and muscle, by coordinating their behavior through communication with other cells. Cell interactions also play essential roles during embryonic development and in basic physiological processes, such as neurotransmission, wound healing, and inflammation. Therefore, the study of cellular interactions is crucial to understand the development and function of multicellular organisms.

Cell–cell interaction is a complex phenomenon; a single cell can interact with many other cells through physical contact, surface receptor-ligand interaction, cellular junctions, and secreted stimulus from neighboring cells or those of distant organs. Interactions via secreted factors such as protein or peptide-based growth factors and cytokines [2], small molecules and metabolites [3] has been extensively studied. More recently, interactions involving extracellular vesicles have emerged as another way of interaction [4]. In addition, cell–cell interactions are affected by their physiological environments, including physical properties of the surrounding extracellular matrix and its biochemical properties, like levels of oxygen (hypoxia) or nutrients (energy deprivation) [5]. Together, these mechanisms of cell interaction contribute to coordinated cellular behavior and complex biological functions in tissues.

Cancer is a major healthcare challenge worldwide. Cell communication in the tumor microenvironment (TME) presents one of the biggest barriers to our understanding of this disease. Tumor tissue is not solely composed of cancer cells; as much as 50% of its composition can derive from non-cancerous cells, along with non-cellular components [6]. Cancer cells interact with themselves and non-cancerous host cells including immune cells (T cells, B cells, macrophages, dendritic cells, NK cells), endothelial cells, and fibroblasts (reviewed in [7, 8] and elsewhere). Cancer cells also respond to growth factors, cytokines, and extracellular matrix proteins present in the TME [9]. The interactions between normal and cancer cells can be tumor-suppressive, i.e. immunoreaction against cancer, but often eventually become tumor-promoting. Immune cells including T cell, B cells, macrophages, dendritic cells, neutrophils, become immune-suppressive and even cancer-supporting, which has been reviewed [10–13]. Cancer-associated fibroblasts are also exploited to secrete extracellular matrix and promote growth and invasion of the cancer cells [14]. Cancer cells interact with endothelial cells to elongate blood vessels in TME [15]. Interactions with extracellular matrix stiffening promotes cancer progression [16]. Overall, interactions within the TME are diverse and promote cancer progression. Therefore, understanding cell–cell interaction within TME will not only provide insight into cancer biology but also the potential for identifying new therapeutics.

In this review, we will discuss the current approaches to the study of cell–cell interactions, challenges associated with these approaches, and the need for better technologies to understand cellular interactions focusing on cell–cell interactions in cancer.

## MODEL SYSTEMS TO STUDY CELL–CELL INTERACTIONS IN CANCER

To systematically understand cell–cell interactions in complex tissue microenvironments, we must be able to: (1) identify specific cell types, (2) observe spatial relation among different cell types, (3) determine the mode of interaction (direct or indirect), (4) measure molecular information in single cells, and (5) measure responses to stimuli. Here, we will discuss model systems and approaches to the study of cell–cell interactions in TME.

**Figure 1:**
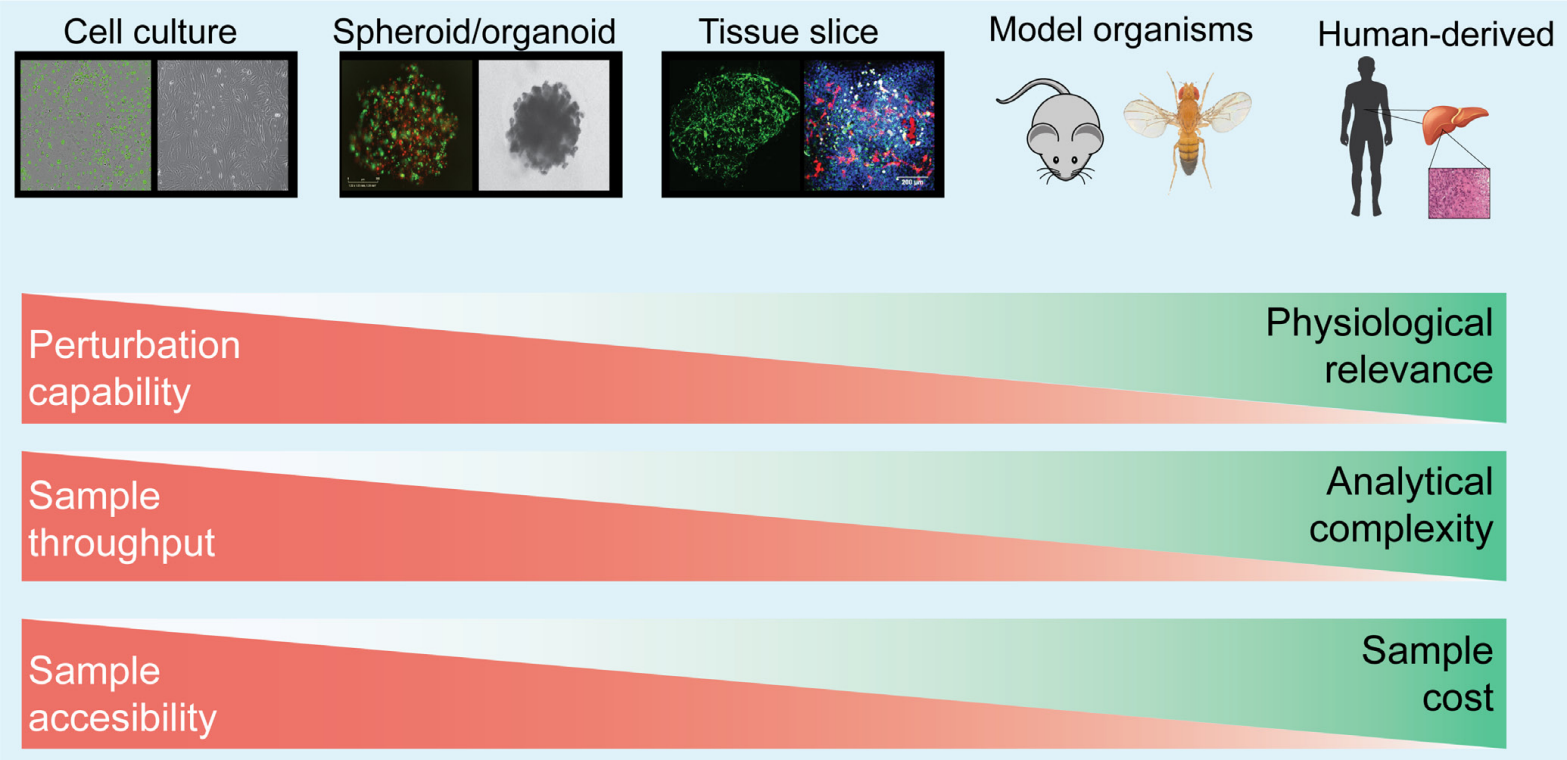
Major experimental model systems for studying cell–cell interaction Model systems ranges from *in vitro* assay of cells grown in monolayer, 3D, and spheroid/organoid cultures, to *in vivo* rodent carcinogenesis models, xenografts of human cancer cell lines, and patient-derived xenografts (PDXs) as well as direct analysis. The strength and weakness of each experimental model are described with red and green bars. A tradeoff exists between sample manipulation and its physiological relevance. Detailed description of each model is also summarized in Table 1.

Many experimental strategies have been developed so far, from *in vitro* assay of cells grown in monolayer, 3D, and spheroid/organoid cultures, to *in vivo* rodent carcinogenesis models, xenografts of human cancer cell lines, and patient-derived xenografts (PDXs) as well as direct analysis of patient samples (Figure 1). A basic tradeoff exists between the ability to manipulate the system and the physiological relevance of the system. Additionally, physiological relevance is often gained at the expense of accessibility and high throughput comprehensive analysis (Figure 1). For cell–cell interaction studies, it is important to select an experimental system appropriate to the purpose of the research. More detailed features of each model system are shown in Table 1.

**Table 1:** Experimental model systems for analyzing cell–cell interactions

	Cell: 2D culture	Cell: 3D culture	Spheroids	Organoids	Tissue slices	Xenografts	PDX	Syngeneic models	Patient tumors
**Physiologicalrelevance**						+++	+++	+++	+++
**Tissue architecture**				+	+++	+++	+++	+++	+++
**Human-derived stromal cells**				++	+++		+		+++
**Tightly packed cells**		+	++	++	++	+++	+++	+++	+++
**Complex cell types**		+	+	++	+++	+++	+++	+++	+++
**Sample availability (reproducibility)**	+++	++	++	+	+	+			
**Molecular intervention**	+++	++	+	+	+	+			

+++ denotes strong relevance; ++ denotes moderate relevance; + denotes low relevance.

### Primary tissues from patient tumors

The most physiologically relevant samples are biopsies and surgical samples obtained from patient tumors. Although they contain all characteristics of the TME, including cellular and non-cellular components, they provide limited accessibility. Most analyses of patient tumors are based on histology. The tumor samples are either fixed or frozen for histological analysis. Analyses of tumor tissue sections from patients exhibit various cell types in native TME, and are often used to assess correlation between histological observations (abundance of various cell types such as macrophages, T cells, extracellular matrix, microvasculature) and clinical outcomes/prognosis [17–19]. Primary tumor tissues are also used to isolate non-cancerous cell population such as cancer-associated fibroblast (CAF). Molecular and phenotypic characteristics of CAFs can be compared to those cells from other region of the body to study how cancer cells can affect their phenotype [20, 21]. However, fixed/frozen tumor tissues cannot be expanded, which limits acquisition of dynamic phenotypic or molecular response. Further, genetic manipulation and comprehensive high-throughput analysis of individual cells are not feasible for frozen/fixed patient tumors. Moreover, patient tumor samples are confounded by genetic background and tissue subtypes, making comparisons between experimental and control groups difficult. Therefore, hypotheses generated from tissue-based results are further tested in *in vitro* culture models.

### Cultured cell line-based *in vitro* models

Cancer cell lines derived from tumor tissues are widely used as 2D monolayer cultures. For cell–cell interaction, co-culture experiments seeding two or more cell lines into the same dish, or seeding into cell culture insert separated by a thin membrane that only allows secreted factors to pass through, are commonly used *in vitro* models. For example, coculture of cancer cells and endothelial cells, fibroblasts and immune cells are often used in various types of cancer to provide insight into cell–cell interaction between cancer and host cells [22, 23]. Cell lines grown *in vitro* are easy to expand and amenable to genetic/pharmacological perturbation studies. However, the biophysical and biochemical properties of cells cultured in monolayer with artificial medium are very different from those of patient tumors where multiple types of cells are tightly packed next each other to form the TME. A cluster of cells limits access to oxygen and nutrients, and poses growth inhibition by contact inhibition. Moreover, cells lines cannot recreate the complex combination of cells and non-cellular components in TME.

To mimic tightly-packed 3D structures of tissues, spheroids can be formed from cultured cells [24]. Spheroids are sphere-like cell aggregates which can be prepared from a single cancer cell type, or mixture of several cell types, e.g. a combination of cancer cells, fibroblasts, and endothelial cells. Spheroid culture gives rise to tight cell junctions and gradients of oxygen and nutrients that more accurately mimic *in vivo* cell growth. Other 3D culture platforms reconstruct TME by mixing cancer cells and non-cancerous cells and providing scaffolds for cells based on natural and synthetic matrices as extracellular matrices [25]. By testing combinations of stromal cells and cancer cells, Wang and colleagues demonstrated the importance of stromal cells to hepatocellular cell malignancy [26]. Although spheroids require more preparation time than monolayer cells, gene modification remains easier than *in vivo*, and high-throughput analysis is becoming increasingly accessible. However, a non-physiological distribution of various cell types and matrix proteins remains a major drawback of this model system. Overall, although *in vitro* culture models are useful for molecular analysis of cell–cell interaction, careful evaluation of these findings in physiological models are often needed [27].

### *Ex vivo* tissue-derived models

To overcome the problems of limited availability of patient tumors, tumors are expanded either *in vitro*, or *in vivo* in mice and other model organisms. Organoids, which are small pieces (∼70 µm) of tissues cultured and expanded in medium, can be prepared from organs such as brains and small intestines, and from tumors as well [28, 29]. Organoids provide the advantage of expansion while preserving the basic histological characteristics of the original source organs [30]. This technology enables testing of multiple experimental conditions on tissues from the same origin. Histological observation and co-culture, chemical or antibody-based perturbation of interaction reveal relationships of cells of interest in tissue-context [31, 32]. In addition, while they are still more difficult than in cell culture, gene introduction and other molecular manipulations are possible [33]. However, passaging of organoids introduces *in vitro* cultivation-driven cell-type specific bias and limits their physiological relevance [34, 35]. Still, organoids are a model that merges TME complexity and *in vitro* convenience.

Another tissue-based model that preserves the TME is precision-cut tumor slices. Tissue slices are thin-cut (200 µm or more) slices of tumor tissues maintained in culture. Live immune cell migration into tumor tissues from pancreatic ductal adenocarcinoma slice cultures was observed using fluorescently conjugated antibodies [36]. Another method of applying tissue slices to TME studies is to co-culture cancer cells into organotypic tissue slices. Mouse brain tissue slice cultured with glioblastoma cells was used as a model to study cellular response of glioblastoma cells to niche signals and drug treatment [37]. Tissue slices have similar benefits to organoids; however, they are not intended to expand, but rather to maintain their viability. While they do not provide the advantage of expansion, tissue slices generate less concern over changing phenotypes during culture.

### *In vivo* tissue-based models

PDX model involves subcutaneous implantation of patient tumor in immune-compromised mice. PDX models allow expansion of human tumors under mouse physiological conditions. As they maintain the original structures of the tumor partially, PDX provides physiologically-relevant models. PDX models have been used for many TME studies, especially to test responses to perturbations of TME in tumor growth and metastasis. Contribution of stromal cell population to aggregate tumor progression has also been studied using PDX models [38, 39]. However, it usually takes more than 1 year to establish them, and strong selection bias including replacement of stroma occurs during establishment and passage [40]. Cancer cell line-derived xenografts (CDX) typically take much less time (1–2 months) to form tumors, but all nonmalignant and non-cellular components are of mouse-origin. A major limitation of both PDX and CDX is the lack of functional immune cells, which play significant roles in the TME. Alternatively, chemical or genetically-induced mouse carcinogenesis models and mouse allograft models can provide tumor tissues under mouse physiological conditions with fully functional immune cells [41, 42]. While studying cell–cell interaction in the TME of mouse tumors can provide critical information on human TME, some key differences between mouse and human immune systems limit enthusiasm for these models.

Overall, using an appropriate experimental system that balances sample accessibility and manipulability with physiological relevance can enable researchers to elucidate cell–cell interactions in complex tissues.

## ANALYTICAL APPROACHES TO STUDY CELL–CELL INTERACTIONS

**Figure 2:**
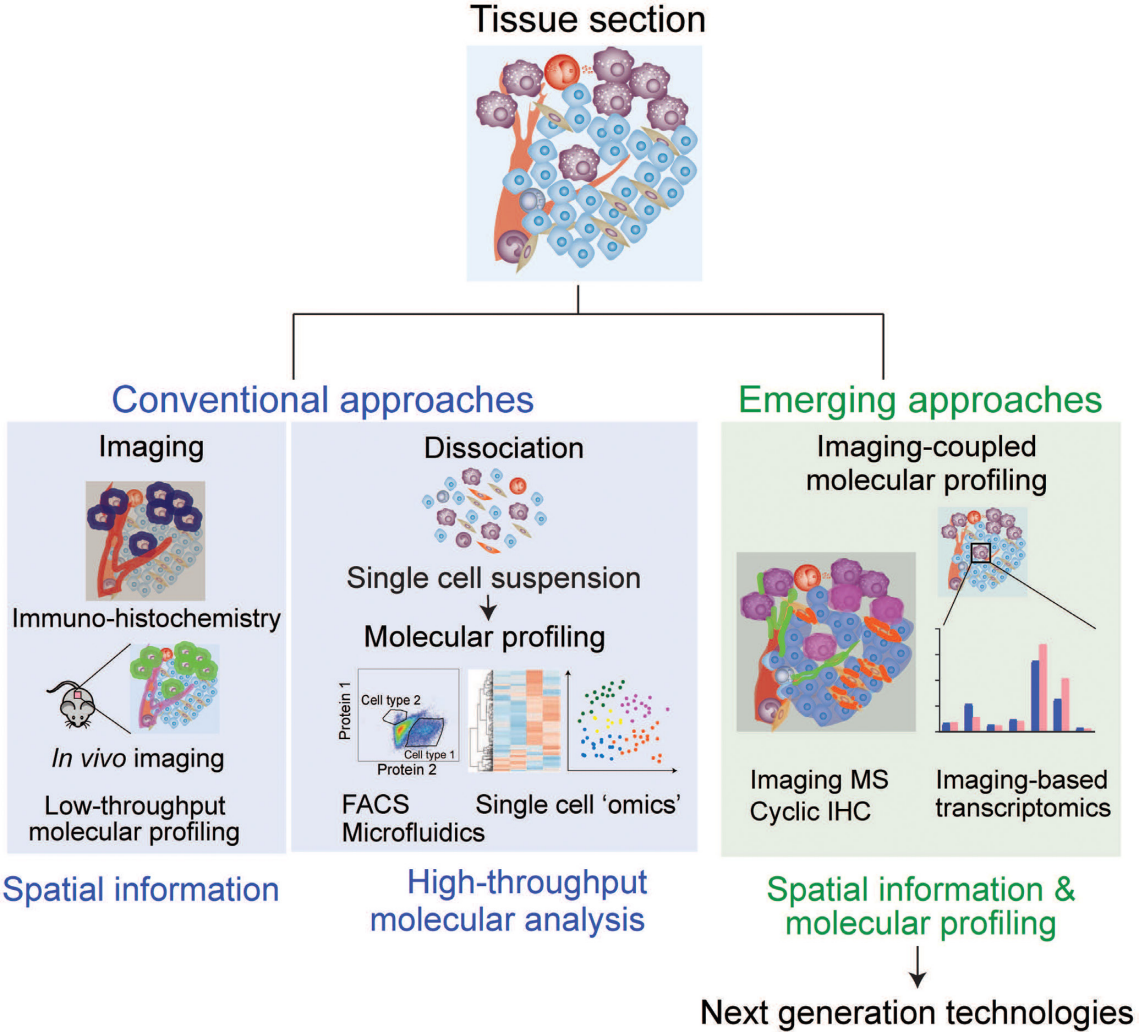
Analytical approaches to study cell–cell interaction Current microscopy-based analyses are focused on obtaining spatial information of targeted cells/molecules, and cell sorting-based techniques require cell dissociation, which precludes spatial information. Emerging approaches integrate both advantages to obtain molecular and spatial information simultaneously, providing meaningful insights for cell–cell interactions.

Using the model systems described above, many analytical approaches have been developed to elucidate cell–cell interactions in complex TME. Here, we describe the current and emerging tools as well as discuss their advantages and weaknesses (Figure 2, Table 2). Currently, many approaches can help obtain either spatial distribution of the cells or detailed molecular information of certain cell types, however, very few approaches can provide both. Therefore, hypotheses generated from one approach are generally tested by combination of other approaches, such as combination of imaging-based strategies and high-throughput analyses of dispersed cells followed by molecular analyses.

**Table 2:** Experimental approaches for analyzing cell–cell interactions

	IHC	Microscopy	*In vivo* imaging	Flow cytometry	Mass cytometry	Microfluidics	(F)ISH	Imaging MS	Raman spec	Cyclic IHC
**Spatial information**	+++	+++	+++	---	---	---	+++	+++	+	+++
**Live cellswithout fixing**	-	++	+++	+	+	++	---	---	+++	---
**Time course live imaging**	-	++	+++			+++			+++	
**Molecular analysis (low throughput)**	+	+	+	++	++	+	+	++	+	++
**Omics analysis (High throughput)**	---	--	-	+++	+++	+++	+	+++	-	--

+++ denotes strong advantage; ++ denotes moderate advantage; + denotes low advantage; - denotes disadvantage.

### Imaging

Imaging is the most powerful tool for visualizing the structure of tissues, spatial distributions of cells, and determining specific cell types. Many microscopy-based imaging approaches have been developed to observe cellular distributions within tissues, such as confocal microscopy, super-high-resolution microscopy, *in vivo* two photon microscopy, electron microscopy and atomic force microscopy. However, quantification and throughput are the two main weaknesses of imaging-based methods. Imaging can quantify proteins, RNAs, lipids and sugar chains using labeled antibodies; however, microscopy-based quantification is easily confounded by many factors including poor signal-to-noise ratio, high background, photobleaching of fluorophore and low resolution of the images [43]. Additionally, the number of targets that can be detected is limited by antibody availability and the number of fluorophores in the visible spectrum. Overall, the imaging allows visualization of cell–cell interactions with less accurate quantification and limited throughput. Recent technological advances, however, have been improving throughput of imaging in high-content imaging and automated microscopes [44].

#### Fixed tissue imaging

Immunohistochemistry (IHC) has been used since the 19th century to directly detect and observe the cells in tissue sections [45]. It is still widely used to observe the structure and distribution of specific cells in tissues in laboratory experiments and in clinical cancer diagnosis [46]. Most of the clinical samples are chemically fixed for further analysis. These histology images have contributed to the vast body of knowledge in understanding the distribution pattern of cells in tumor tissues. Cells labeled by cell type-specific dyes and antibodies enable the gain of spatial information in the tissue. Observation of tissue section showed disseminated breast cancer cell in proximity of microvasculature are maintained in dormant state [47]. Relative protein expression levels can also be quantified using IHC. Further, laser capture microdissection systems can directly excise a specific area or single cells for downstream analysis of global gene expression and proteomic analysis, providing both spatial information and correlated molecular information [48]. However, despite its widespread use, IHC has some drawbacks. First, fixation precludes time-scale analysis on live cells. Second, fixation of tissues lowers the quality of RNA, DNA and proteins extracted from sample tissue, which poses major obstacles to comprehensive analysis of limited samples available from a section. Although throughput has improved with tissue microarrays that contain up to 1000 tissues on one slide, analysis time per sample is limited by microscope observation.

#### Live tissue imaging

To overcome limitations posed by tissue fixation, recent technologies have been developed to enable high-resolution, real-time imaging under more physiological states, either *ex vivo* or *in vivo* [49]. Tissue architecture including detailed morphologies and distributions of individual cells are visualized in *ex vivo* tissues by labeling using fluorescent-labeled dyes, antibodies, and introduction of fluorophores to target proteins. Recently, the advent of CRISPR-Cas9-mediated cell-type specific labeling has enabled live cell tracking (lineage tracing) and studies of cell–cell interactions [50–53]. Several approaches to *in vivo* imaging have also emerged that allow imaging of cell–cell interactions under the most physiological conditions [54]. Intravital microscopy, imaging of live animal at microscopic resolution, is a strong tool to observe cell behavior directly, including cell migration and direct contacts [55]. However, imaging depth (∼1000 µm, mostly a few to hundreds µm) [49] and challenges in *in vivo* labeling of specific cell-type limit the application of these methods. Alternatively, whole body distribution of disseminated cancer cells can be detected using *in vivo* imaging techniques such as fluorescence, luminescence, ultrasonography (US), positron emission tomography (PET), magnetic resonance imaging (MRI), and computed tomography (CT) [56]. Because this constitutes a noninvasive investigation, spatial information of TME can be observed repeatedly with time sensitivity under physiological conditions. The weakness is these imaging strategies have lower resolution than *in vivo* microscopy (a few mm). However, Vacok and colleagues applied improved optical coherence tomography to mouse to achieve three-dimensional, wide-field, in-depth (2 mm) live observations of blood vessel arrangement and cancer necrosis, and drug response of cancer [57].

### Single cell analyses by fluidics

#### Flow cytometry

Flow cytometry can analyze individual cells quantitatively and in high throughput. Developed in the 1960s [58], it became an indispensable technology for analysis of mixed cell populations. One classical usage for cell interaction studies is to detect direct physical cell–cell interactions in high-throughput manner [59–61]. Multiplex labeling identifies composition of various cell types that exist in TME based on surface protein marker expression [62]. Changes in cell components can provide insight into the role of stromal cells in tumor-supporting microenvironment [63, 64]. In addition, quantification of multiple proteins in single cells using dyes or fluorescently-labeled antibodies can be achieved using flow cytometry. Similarly, flow cytometry has also been used to measure mRNA or microRNA expression levels using labeled antisense nucleotides [65, 66]. In combination with Fluorescence Activated Cell Sorting (FACS), target cells can be isolated and collected in a high-throughput manner, enabling further comprehensive gene expression and proteomic analysis. For example, tumor-infiltrated immune cell populations can be classified and collected for further molecular analysis [67]. Imaging flow cytometry is another high throughput approach to analyze thousands of individual cells per second by taking images of cells simultaneously to provide morphological information [68]. This provide advantage over traditional flow cytometry in multiplexing imaging capability. High-throughput analysis of flow cytometry has enabled studies of rare cell types, such as cancer stem cells. FACS can analyze and sort cancer stem cell-like population from tumor for further investigations. For example, stem cell-like cells in patient-derived glioblastoma specimen were isolated as a EGFR-positive population for transcriptome analysis [69].

However, flow cytometry and other single cell-based methodologies have notable drawback that requires tissue dissociation and single cell suspension. Collagenase and/or trypsin are used to disperse cells, which degrade adhesion molecules, tight junctions and extracellular matrices in TME. Information on three-dimensional distributions of cells and non-cellular components is completely lost during this process. Therefore, although flow cytometry has strong advantages for molecular analysis, it cannot obtain spatial and structural effects on cell–cell interaction simultaneously.

#### Microfluidics

Microfluidics offers another way to analyze cells in a high throughput manner at single cell levels, with a notable difference from flow cytometry, by culturing single cells in a microchamber. Single cells are isolated by trapping the cells in a microfluidics cavity, then the cells are incubated and monitored real-time, or proteins and RNA levels can be analyzed directly on chip [70]. Wang and colleagues directly observed effect of cell interaction of glioblastoma multiforme cancer cells in controlled microchip [71]. Recently-developed “cancer-on-a-chip”, where cancer cells are cultured in microchambers mimicking TME, provide insight into interaction between cancer cells and TME [72, 73]. Similarly, the organ chip model mimicking lung environment with lung cancer cells has been shown to recapitulate growth rate, therapeutic responses and metastatic niche [74]. These microchambers allow observation of real-time and 3D immune-cancer cell interactions [75], cell behavior in metastatic cascades, such as EMT, invasion and adhesion for intra-and extravasation [76]. However, the requirement of tissue dispersion to single cells and difficulties in mimicking TME *in vitro* remain a challenge.

Overall, flow cytometry followed by FACS and microfluidics are ideal for high throughput comprehensive molecular analysis. However, the loss of structural information to cell dispersion required for the techniques remains as a critical issue.

### Molecular analyses of single cells

#### Transcriptomic analysis: single cell-RNA sequencing (sc-RNAseq)

Recent droplet-based technological advances have enabled transcriptomic profiling of single cells [77, 78]. Cells dissociated from tissues were isolated into single cells by FACS, microfluidics, microdroplets or picowells to capture single cell per a droplet or a well [79]. Droplet-based technology integrated with sc-RNAseq has been applied to profile intra-tumoral immune cells in breast cancer [80]. Patel and colleagues transcriptionally described heterogeneity in glioblastoma using sc-RNAseq [81]. Combined with DNA barcoding, thousands of individual cells can be now analyzed [78]. The use of single cell-RNA sequencing is being widely adopted to describe heterogenous population in complex tissues.

#### Protein analyses: mass cytometry

Proteomic analysis in single cell is in need because transcription and actual protein amount is often different. While comprehensive analyses of proteins are not yet possible, mass cytometry is one of the most high-throughput protein analyses. Isolated cells are labeled with multiplexed metal-conjugated antibodies, and the amount of target proteins is detected by Cytometry by Time of Flight (CyTOF) mass spectrometry [82]. The current technology enables to measure approximately 40 proteins simultaneously [83]. Simoni et al recently described CD8^+^ T cell populations infiltrated into different types of tumors [84].

Although dissociation of the tissue disrupts spatial information, high-throughput molecular analyses in single cell level boost generation of hypothesis relating to cell–cell interactions that are subjected to validation using other experimental approaches.

## EMERGING APPROACHES TO ANALYZE CELL–CELL INTERACTIONS

To study cell–cell interaction in TME, spatial information at the resolution of a single cell is essential, and microscopy is currently the most powerful tool to obtain it. However, current imaging technologies are intractable to comprehensive molecular analysis. To further understand complex cell–cell interactions, technologies that offer both spatial information and high-throughput molecular analyses are highly needed. Here, we discuss some emerging approaches that have been developed to obtain molecular information linked with spatial information (Figure 2).

### Imaging-coupled transcriptional profiling

The importance of connection between transcription and spatial distribution of the cells are discussed in other reviews [85, 86]. Fluorescent *in situ* hybridization (FISH) allows measurement of gene expression using fluorescent probes recognizing RNAs linking transcription patterns with spatial organization of single cell cells within tissues. FISH usually has low throughput due to limits on the number of fluorescent probes that can be used simultaneously. Recently, multiplexed *in situ* hybridization has been developed. Moffitt and colleagues developed multiplexed *in situ* hybridization that can analyze 130 RNAs from approximately 40,000 cells in 18 h, using error-robust barcodes and sequential cleavage of fluorophores [87]. Although this strategy dramatically enhances the throughput of FISH, the number of transcripts that can be measured simultaneously is still far from comprehensive analysis. Wang and colleagues developed 3D *in-situ* RNA sequence, named STARmap, using mouse brain [88]. They could analyze up to 1020 marker genes in single cell level, and categorized cells into 16 cell types based on expression patterns. The *in situ* transcription analyses outlined above suffer from the need for tissue fixation. A sequence-based strategy overcomes this by introducing photoactivatable tags into live cells together with a cell penetrating peptide [89]. Activation of these tags within specific target cells allows isolation of mRNA signals from individual cells for transcriptomic analysis. This strategy has advantages that allow for analysis of live cells and use of well-established RNA-seq platforms. Overall, imaging-based transcriptome will describe the cells in different transcription state related to its surrounding environment.

### Imaging-based mass spectrometry (IMS)

IMS is an approach that integrates the strength of imaging and unbiased proteomic analysis through matrix-assisted laser desorption ionization [90]. By measuring mass/charge signals at various coordinates, it can analyze both the existence of target molecules and information about their localization in tissues. IMS enables analysis of thousands of proteins, lipids, and metabolites without labeling directly from the tissues. Despite these advantages, IMS suffers from two main drawbacks: 1) low resolution (5–200 μm for IMS vs 1 μm for fluorescence microscopy); and 2) low sensitivity that causes inability to detect low abundant proteins. To enable detection of low abundant or active form of proteins, a combination of metal-labeled antibodies and mass cytometry has been developed [91]. Using rare earth metals-labeled antibodies, it is possible to detect at least 32 (and possibly up to 100) proteins using more antibodies. Giesen and colleagues have developed high resolution imaging mass cytometry by combining IHC imaging and laser-based ablation for mass cytometry [92]. Mirnezami and colleagues could show heterogeneity of patient colorectal cancer by lipid-based analysis [93]. It has been shown that sequential images can be used to develop 3D IMS image [94]. These imaging technologies have improved resolution and provide single cell level analyses, however, there is still a tradeoff between resolution and comprehensive analysis of molecules.

### Raman microscopy (RM)

Raman spectroscopy (RS), an optical technique based on inelastic scattering of light by vibrating molecules, has been adapted to provide chemical fingerprints of cells in complex tissues. Raman microscope (RM), a microscope coupled with RS, can elucidate relationships between tissue architecture and molecular distributions. Studies using RM to aid clinical diagnosis have been reported [95, 96]. RM is non-invasive and requires no staining or labeling, which permits dynamic acquisition of molecular data such as lipids, proteins, and water content in live tissues. RM has been used for identifying lipid composition in TME [97]. In combination with 2-photon microscopy, Lee and colleagues imaged invasive cancer cells, blood flow, and direct cancer cells-host cells interaction in TME of mouse mammary tumors [98]. Inability to provide molecular (genomic and proteomic) information limits the usefulness of this emerging approach.

### Cyclic immunofluorescence (IF)

IHC is limited by the number of antibodies that can be used on a single section. This stems from the requirement that antibodies from different species be used for each target, and that most microscopes only detect <10 fluorescent channels. To overcome these limitations, several strategies to inactivate, block, or strip the first round of antibodies to allow multiple rounds of IHC on a single section have been developed [99, 100]. Lin and colleagues optimized 3-color cyclic IF to perform multiplexed analyses of drug response of melanoma cells with 5 cycles of cyclic IF [101]. Cyclic IF has also been shown to be effective on tissue samples [102]. Although antibody staining cycles are theoretically unlimited, this strategy is practically limited to approximately 16 channels because of damage to samples and long per-round staining time resulting in low throughput. Therefore, cyclic immunofluorescence has methodological limitation to be applicable for more comprehensive analyses.

## CONCLUSIONS

The structural and functional integrity of all multicellular organisms is coordinated by and dependent on cell–cell communication mediated via spatial and temporal interaction. The study of cell–cell interactions is crucial to the understanding of many biological processes, including organ formation and size control, tissue packing, and immunological reactions. Without cell–cell contact, our tissues, organs and whole body would lose their structure and function; even slight disturbances can result in pathological conditions such as cancer. It is widely recognized that cell–cell interactions in the microenvironment contribute to tumor progression. In order to study a phenomenon as complex as cell–cell interaction, careful experimental designs using relevant model systems and analytical strategies must be implemented.

Using cell–cell interactions in the tumor tissue as an example, we first discussed various experimental models that are used to study TME. In general, there is a tradeoff between applicable experimental strategies and physiological relevance of the system. Understanding the limitations and exploiting the advantages of each experimental model are fundamental to this research. Current experimental approaches provide either high-throughput molecular analysis (cell sorting-based methods) or spatial information through imaging (microscopy-based methods). Emerging approaches combine the advantages of these two to perform both spatial distribution analysis and high-throughput molecular analysis (Figure 2). A combination of microscopy and ‘omics’ (ex, tagged RNAseq and IMS) will provide a powerful strategy for investigating cell–cell interactions.

Recent technical advances have enabled single cell transcriptomics (scRNAseq); for biochemists and molecular biologists, however, high throughput single cell proteomic analysis remains a challenge. Proteins are the actual effectors of cellular functions, but transcription levels and protein levels do not correlate in many cases. Therefore, highly sensitive MS instruments that can analyze large number of proteins from a single cell are required.

Further, methods that will allow delivery of gene modification (knockdown/overexpression) to targeted single cells in tissues in a high throughput manner and enable dynamic/kinetic measurements are also needed. Application of basic molecular biology techniques to specific cell-type of interest in a complex tissue context will be a strong tool to reveal the effect of one cell on the surrounding cells. In addition, to reconstitute the regulatory networks, we will need to elaborate computational modeling strategies that combine all molecular and spatial information data [103].

Cells interact through both transient and prolonged physical contact, surface receptor interaction, cellular junctions, cell matrix interactions, and secreted stimuli from neighboring cells or from distant organs. Can we determine the type of cell interactions, whether it is direct contact, autocrine or paracrine signaling? New approaches are also needed to answer some of these fundamental questions in cell communication.

Overall, new technologies, together with appropriate experimental models, will reveal the spatial and molecular resolution of cell–cell interaction. Insight into cell–cell interaction will provide deeper understandings of TME as well as the physiological systems of multicellular organisms.
